# NLRP3 inflammasome triggers interleukin‐37 release from human monocytes

**DOI:** 10.1002/eji.202149724

**Published:** 2022-04-28

**Authors:** Anna Gritsenko, Rodrigo Díaz‐Pino, Gloria López‐Castejón

**Affiliations:** ^1^ Lydia Becker Institute of Immunology and Inflammation, Faculty of Biology, Medicine and Health University of Manchester Manchester UK; ^2^ School of Biological Sciences, Division of Infection, Immunity and Respiratory Medicine, Faculty of Biology, Medicine and Health University of Manchester, Manchester Academic Health Science Centre Manchester UK

**Keywords:** monocytes, NLRP3, caspase‐1, gasdermin, interleukin‐37

## Abstract

IL‐37 is an anti‐inflammatory member of the IL‐1 family that dampens inflammation associated with many noncommunicable diseases. However, mechanisms of IL‐37 regulation remain understudied. We aimed to investigate the enzymatic cleavage of IL‐37 that potentiates extracellular signalling, as well as pathways of IL‐37 secretion. In human monocytes, mature IL‐37 (mIL‐37) was released following canonical NLRP3 inflammasome activation. The release of IL‐37 was blocked by inhibiting plasma membrane permeability and in gasdermin‐D‐deficient THP‐1 cells. While the cleavage of IL‐37 was found to be constitutive, the release of mIL‐37 was blocked in NLRP3‐deficient THP‐1 cells and by NLRP3 inhibitor MCC950 in THP‐1s and primary human monocytes. IL‐37 secretion also occurred after 18‐h exposure to LPS, independently of the alternative NLRP3 inflammasome. This LPS‐dependent IL‐37 secretion required plasma membrane permeability, but not conventional protein secretion apparatus. Mutagenesis of the suggested caspase‐1 cleavage site (D20) or the proposed alternative cleavage site (V46) did not completely block IL‐37 processing. Therefore, we propose a novel pathway in which IL‐37 is cleaved by caspase‐1‐independent mechanisms and released following canonical and alternative NLRP3 inflammasome triggers by differential pathways.

## Graphical abstract

**A**. In human monocytes, IL‐37 cleavage occurs constitutively but not at positions D20 or V46. **B. **Long‐term LPS stimulation induces mature IL‐37 release independently of alternative NLRP3 inflammasome but dependent on membrane permeability. **C. **Mature IL‐37 release is mediated by canonical NLRP3 inflammasome activation, gasdermin‐D, and membrane permeability.

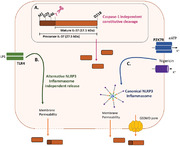

## Introduction

IL‐37 is a member of the IL‐1 family of cytokines that was first discovered using *in silico* methods in human cells [1, 2]. Although IL‐37 is well conserved in primates [3], there is no known homologue in mice. IL‐37 has anti‐inflammatory functions that suppress an overactive immune response seen in many inflammatory conditions, but the mechanisms of its regulation remain poorly studied. Alternative splicing of the IL‐37 mRNA generates different combinations of exons, producing five isoforms, named IL‐37a‐e [4]. IL‐37b is the most abundant and best characterized isoform. Within human PBMCs, monocytes are the main producers of IL‐37 [5]. IL‐37 signals through binding to the IL‐18 receptor subunit IL‐18Rα and recruiting the decoy receptor IL‐1R8 [6]. IL‐1R8‐deficient mice are not able to downregulate TNF‐α and IL‐6 genes in response to IL‐37 [6]. Although both IL‐37 precursor (pro‐IL‐37) and cleaved forms (mIL‐37) are biologically active, IL‐37 lacking the first 20 amino acids (expression starting at predicted D20 caspase‐1 cleavage site) has higher anti‐inflammatory potency [7]. Indeed, cleaved IL‐37 at this site has been reported to bind to soluble IL‐18Rα‐Fc with a higher affinity than pro‐IL‐37, potentially resulting in more potent anti‐inflammatory signaling [8].

Kumar et al. (2002) first suggested that caspase‐1 may play a role in IL‐37 processing by showing that recombinant IL‐37 appears cleaved by recombinant caspase‐1 [8]. Secretion of cleaved IL‐37 in response to bacterial LPS stimulation has been described in RAW cells expressing human pro‐IL‐37 [9]. Mutation of the predicted caspase‐1 cleavage site at Asp20 (D20A) in this experimental setting resulted in reduced secretion of this cleaved form [9]. As an effector enzyme of the NOD‐like receptor pyrin domain‐containing protein 3 (NLRP3) inflammasome complex, caspase‐1 plays an important role in the maturation of IL‐1β and IL‐18 cytokines in response to a range of PAMPs or damage‐associated molecular patterns (DAMPs), such as nigericin toxin, extracellular ATP, and lysosomal destabilization agents, for example, silica and cholesterol crystals [10]. Indeed, ATP stimulation of RAW macrophages causes release of cleaved IL‐37 that is reduced by treatment with a pan‐caspase inhibitor. ATP‐dependent release of cleaved IL‐37 is also observed from PBMCs [9]. However, whether release of mIL‐37 was due to cleavage by caspase‐1 directly, or as a consequence of downstream events triggered by inflammasome assembly was not investigated. An alternative cleavage of IL‐37 at Val46 (V46) has been reported using COS7 cells overexpressing IL‐37, although the protease responsible for this cleavage remains unknown [11]. Moreover, IL‐37 can also feedback to suppress further inflammasome activation as treatment of murine Bone Marrow Derived Macrophages (BMDMs), with this cytokine inhibits Apoptosis‐associated speck‐like protein containing a CARD (ASC) oligomerization, caspase‐1 activation, and IL‐1β and IL‐18 secretion [12].

At present, little is known about how IL‐37 is secreted from the cell. Previous work suggested that IL‐37 is released via the classical ER‐Golgi protein secretion pathway in response to LPS stimulation of TLR‐4 on human monocytes [5]. Whether this is the precursor or the cleaved form was not investigated. Long‐term exposure to LPS is known to initiate an alternative mechanism of NLRP3 activation and IL‐1β release in monocytes that is independent of potassium ion efflux and cell death [13]. However, the involvement of the alternative NLRP3 inflammasome in IL‐37 release remains uncharacterized. Moreover, it is widely accepted that IL‐37, like IL‐1β, does not contain a signal peptide to target it for secretion via the ER‐Golgi pathway [14]. Indeed, it has been reported that mature IL‐37 (mIL‐37), like other leaderless proteins, for example, mIL‐1β, enters the ER‐Golgi intermediate compartment (ERGIC) via interaction with TMED10 [15]. With the aid of HSP90B1, the TMED10 channel translocates cargo into the lumen of the ERGIC, facilitating its release. Another pathway for the release of leaderless proteins like IL‐1β and IL‐18 is through caspase‐1‐mediated cleavage of gasdermin‐D (GSDMD). The generation of GSDMD *N*‐terminal fragment allows lytic pore formation, resulting in pyroptotic cell death and the secretion of mature IL‐1β and IL‐18 [16].

This study aimed to unravel the mechanisms of IL‐37 cleavage and release in primary human monocytes, focusing on the role of canonical and alternative NLRP3 inflammasomes and consequent GSDMD‐driven pyroptosis.

## Results

### NLRP3 inflammasome activation causes the release of IL‐37

Primary human monocytes and THP‐1 cells were utilized in this study to investigate the mechanisms of endogenous IL‐37 regulation in inflammasome‐forming cells. Alternative splicing generates five isoforms of IL‐37 [17]. To confirm which isoforms are present in these cells, we initially used quantitative PCR using primers specific to splice junctions as indicated in Supporting information Fig. S1A. In resting and LPS‐stimulated monocytes (Supporting information Fig. S1B) and THP‐1 cells (Supporting information Fig. S1C) isoform B showed the highest level of expression compared to all other isoforms and was upregulated by LPS stimulation.

**Figure 1:**
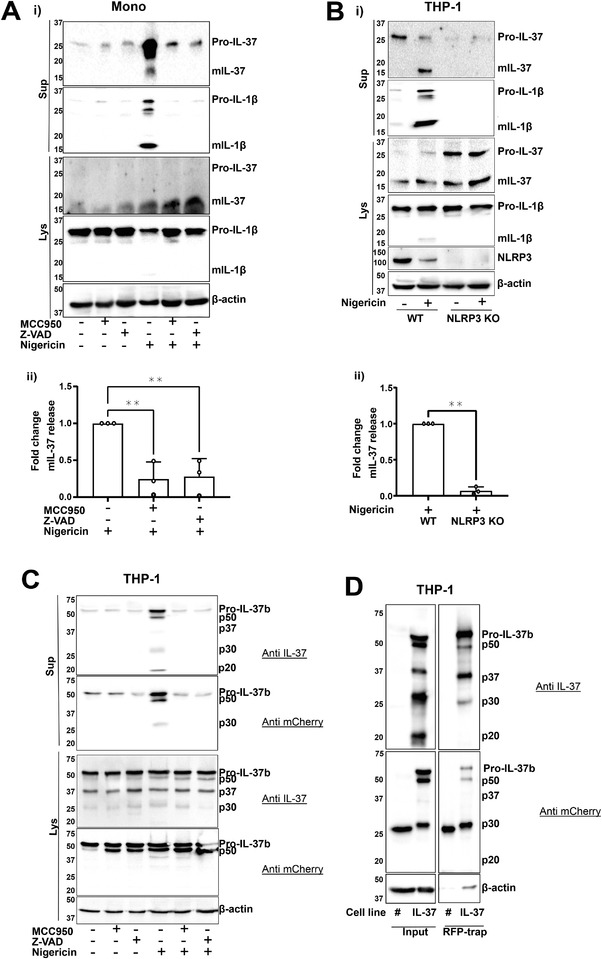
**NLRP3 inflammasome activation mediates the release of IL‐37**. (A) Primary human monocytes were primed with LPS (1 μg/mL, 4 h), preincubated with MCC950 (1 μM) or Z‐VAD (50 μM) for 15 min prior to treatment with nigericin (10 μM, 45 min). (Ai) Western blot analysis was carried out for mIL‐37 (17.5 kDa), pro‐IL‐37 (27.5 kDa), mIL‐1β (17 kDa), pro‐IL‐1β (31 kDa), as well as loading control β‐actin (42 kDa). (Aii) Levels of mIL‐37 release were quantified using densitometry and standardized to mIL37 release after nigericin alone treatment. n = 3. **p < 0.01 using one‐way ANOVA. (B) WT or NLRP3 KO THP‐1 were primed with LPS (1 μg/mL, 4 h) and treated with nigericin (10 μM, 45 min). (Bi) Western blot analysis was carried out for mIL‐37 (17.5 kDa), pro‐IL‐37 (27.5 kDa), mIL‐1β (17 kDa), pro‐IL‐1β (31 kDa), NLRP3 (113 kDa), as well as loading control β‐actin (42 kDa). (Bii) Levels of mIL‐37 release were quantified using densitometry and standardized to nigericin alone treatment. n = 3. **p < 0.01 using *t*‐test. (C) IL‐37b‐mCherry expressing THP‐1 cells were preincubated with MCC950 (1 μM) or Z‐VAD (50 μM) for 15 min prior to treatment with nigericin (10 μM, 45 min). (D) Cherry alone (#mCherry) or IL‐37b‐mCherry THP‐1 cells were stimulated with nigericin (10 μM, 45 min). The supernatant was concentrated, and RFP‐trap immunoprecipitation was carried out. Input and immunoprecipitated fraction were ran on SDS‐PAGE gel. Western blot analysis was carried out for IL‐37b‐mCherry using anti‐IL‐37 antibody (R&D). Blots are representative of at least three independent biological experiments. Data are shown as mean ± SD.

As a caspase‐1 cleavage site on IL‐37b has been described, we aimed to investigate the role of the NLRP3 inflammasome in the processing and release of IL‐37. Primary human monocytes were LPS primed (1 μg/mL, 4 h) and then pretreated with NLRP3 inhibitor MCC950 (1 μM, 15 min) or an irreversible pan‐caspase inhibitor Z‐VAD‐FMK (Z‐VAD, 50 μM, 15 min) prior to stimulation with the NLRP3 inflammasome activator nigericin (10 μM, 45 min). While nigericin caused the maturation and release of IL‐1β, constitutive IL‐37 cleavage, resulting in a loss of 10 kDa from the 27.5 kDa pro form, was observed in the lysates of untreated monocytes (Fig. 1Ai). Moreover, the release of the pro form also occurred constitutively, as previously described from transfected CHO and HEK293 cells [11] and PBMCs [9]. However, the release of mIL‐37 was only initiated by nigericin treatment and inhibited by both MCC950 and Z‐VAD (Fig. 1Aii), thus, revealing the role of NLRP3 and effector caspases in the release of mIL‐37. A similar pattern of release of the smaller, processed form of IL‐37 was observed in THP‐1 cells (Fig. 1Bi) and was detected by two different anti‐IL‐37 antibodies (R&D and Proteintech) (Supporting information Fig. S1D,E). To genetically confirm the involvement of NLRP3, NLRP3 KO THP‐1 cells were used. As expected, mIL‐1β was secreted from LPS primed and nigericin‐activated WT but not NLRP3‐deficient THP‐1 cells (Fig. 1Bi). Similarly, mIL‐37 was not secreted from NLRP3 KO cells (Fig. 1Bii), although the processing was still observed in the lysate. Other NLRP3 inflammasome activators, such as extracellular ATP and cell swelling induced by hypotonicity [18], also enhanced the release of cleaved IL‐37 in addition to mIL‐1β (Supporting information Fig. S2).

To confirm that IL‐37 isoform B specifically is constitutively cleaved and released following inflammasome activation, we utilized THP‐1 stably expressing IL‐37b‐mCherry that appeared at 60 kDa. This experiment was carried out in the absence of priming, which we have previously shown to be dispensable for NLRP3 inflammasome activation in human monocytes and THP‐1 cells [19]. Indeed, *N*‐terminal 10 kDa processing of pro‐IL‐37‐mCherry results in 50 kDa fragment that is detected by both anti‐IL‐37 and anti‐mCherry antibodies. This occurred even in the absence of nigericin and was not inhibited by Z‐VAD, suggesting caspase‐ independent cleavage. Upon nigericin treatment, multiple fragments (p50, p37, p30, and p20) were released into the supernatant (Fig. 1C). The release of all these fragments was repressed by the NLRP3 inhibitor MCC950 and pan‐caspase inhibitor Z‐VAD. To confirm that these fragments were IL‐37, red fluorescent protein (RFP)‐trap was used to pull down IL‐37b‐mCherry from the supernatants of these cells, following nigericin stimulation (10 μM, 45 min). These fragments were separated on a gel, excised, and mass spectrometry analysis was carried out. All fragments were identified as IL‐37, suggesting that IL‐37‐mCherry was cleaved in multiple places (Fig. 1D). However, the specific cleavage sites could not be detected due to low *N*‐terminal coverage. While cleavage of p50, p37, p30 was constitutive, the 20 kDa fragment was only generated upon nigericin stimulation and detected by the IL‐37 but not the mCherry antibody. Moreover, p20 was not pulled down by RFP‐trap which further suggests that C‐terminal processing is cleaving mCherry from IL‐37b or that multiple rounds of processing are taking place to generate the p30 and p20 fragments from the p50 protein.

### IL‐37 release is mediated by lytic cell death and membrane permeability

**Figure 2:**
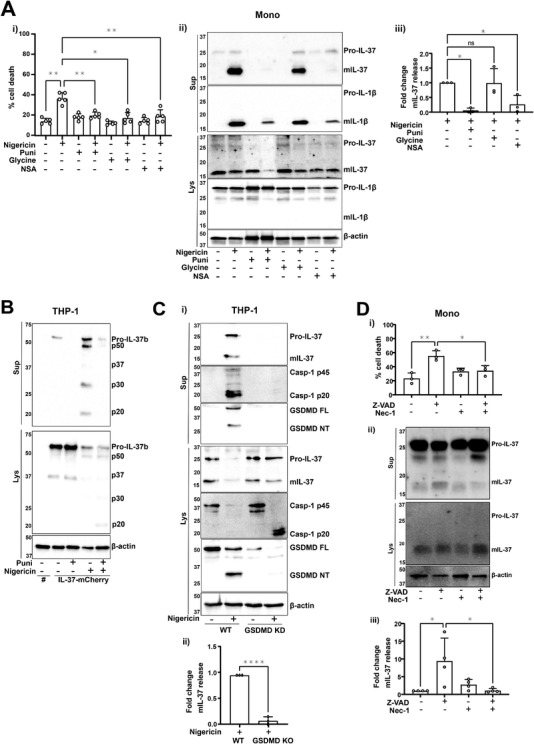
**IL‐37 release is mediated by lytic cell death**. (A) Primary human monocytes were primed with LPS (1 μg/mL, 4 h), preincubated with punicalagin (50 μM), glycine (5 mM), or NSA (20 μM) for 15 min prior to treatment with nigericin (10 μM, 45 min). (Ai) Cell death was measured by LDH assay and shown as percentage relative to total cell death, n = 5. *p < 0.05; **p < 0.01 using one‐way ANOVA. (Aii) Western blot analysis for mIL‐37 (17.5 kDa), pro‐IL‐37 (27.5 kDa), mIL‐1β (17 kDa), pro‐IL‐1β (31 kDa), as well as loading control β‐actin (42 kDa). (Aiii) Levels of mIL‐37 release were quantified using densitometry and standardized to nigericin alone treatment. n = 3. **p < 0.01, ns = not significant using one‐way ANOVA. (B) mCherry alone (#mCherry) or IL‐37b‐mCherry expressing THP‐1 cells were pretreated with punicalagin (50 μM, 15 min) prior to treatment with nigericin (10 μM, 45 min). Anti‐IL‐37 antibody (R&D) was used to detect IL‐37b‐mCherry. (C) WT and GSDMD KO THP‐1s were primed with LPS (1 μg/mL, 4 h) and treated with nigericin (10 μM, 45 min). (Ci) Western blot analysis for mIL‐37 (17.5 kDa), pro‐IL‐37 (27.5 kDa), mIL‐1β (17 kDa), pro‐IL‐1β (31 kDa), mCaspase‐1 (20 kDa), pro‐Caspase‐1 (45 kDa), GSDMD full length (FL, 53 kDa), GSDMD N‐terminus (NT, 31 kDa), as well as loading control β‐actin (42 kDa). (Cii) Levels of mIL‐37 release were quantified using densitometry and standardized to nigericin treatment of WT cells. n = 3. ****p < 0.0001 using *t*‐test. (D) Primary human monocytes were primed with LPS (1 μg/mL, 4 h), preincubated with Nec‐1 (50 μM) for 15 min prior to treatment with Z‐VAD (50 μM, 4 h). (Di) Cell death was measured by LDH assay and shown as percentage relative to total cell death, n = 3. * *p* < 0.05; **p < 0.01 using one‐way ANOVA. (Dii) Western blot analysis was carried out for mIL‐37 (17.5 kDa), pro‐IL‐37 (27.5 kDa), as well as loading control β‐actin (42 kDa). (Diii) Levels of mIL‐37 release were quantified using densitometry and standardized to untreated control. n = 4. *p < 0.05 using one‐way ANOVA. Blots are representative of at least three biologically independent experiments. Data are shown as mean ± SD.

As plasma membrane permeability is linked to IL‐1 release, and is closely associated with pyroptosis, we next asked if mIL‐37 release was mediated by membrane permeability and cell membrane rupture. To address this, primary human monocytes were pretreated with the plasma membrane stabilizing agent punicalagin [20] (50 μM, 15 min), the cytoprotective agent glycine (5 mM, 15 min), or necrosulfonamide (NSA) (20 μM, 15 min): a GSDMD inhibitor [21] that we have previously shown blocks GSDMD pore formation in human cells [22]. Punicalagin, glycine, and NSA reduced the levels of nigericin‐induced cell death (Fig. 2Ai). Punicalagin and NSA, but not glycine, inhibited IL‐1β release, which reflects previous reports describing IL‐1β passing through GSDMD pores independently of membrane rupture that is blocked by glycine [23, 24] (Fig. 2Aii). Similarly, only punicalagin and NSA lowered the level of both pro‐ and mIL‐37 secretion, suggesting that like IL‐1β, IL‐37 may be released through GSDMD pores (Fig. 2Aiii). Release of isoform B specifically via plasma membrane permeability was confirmed to be true by using IL‐37b‐mCherry expressing THP‐1 cells, where punicalagin inhibited the release of pro‐IL‐37b‐mCherry as well as the processed fragments following inflammasome activation (Fig. 2B). The involvement of GSDMD was further investigated. Following nigericin stimulation (10 μM, 45 min) of LPS‐primed THP‐1 cells, inflammasome activation occurred but caspase‐1 p20 was not able to be released in GSDMD KO cells (Fig. 2Ci). mIL‐37 release was detected from WT but not GSDMD‐deficient cells, suggesting that GSDMD induced pyroptosis is mediating IL‐37 release following inflammasome activation (Fig. 2Cii).

As pyroptosis appeared to be a mechanism for mIL‐37 secretion in the context of inflammasome activation, it was next investigated whether other mechanisms of lytic cell death, in the absence of caspase‐1 activation, can also facilitate mIL‐37 release. To address this, necroptosis was induced. Primary human monocytes were LPS primed (1 μg/mL, 4 h), followed by pretreatment with RIPK1 inhibitor necrostatin‐1 (Nec‐1, 50 μM, 15 min). Pan caspase inhibitor Z‐VAD (50 μM, 4 h) was then used to induce necroptosis through caspase‐8 inhibition [25]. Monocytes displayed a significant increase in cell death following Z‐VAD treatment (Fig. 2Di) as well as significant mIL‐37 release (Fig. 2Dii, iii) both of which were reversed by Nec‐1 treatment. This suggests cell death mechanisms involving lytic pore formation can lead to the release of IL‐37.

### Long‐term LPS treatment leads to IL‐37 release independent of the alternative NLRP3 inflammasome

**Figure 3:**
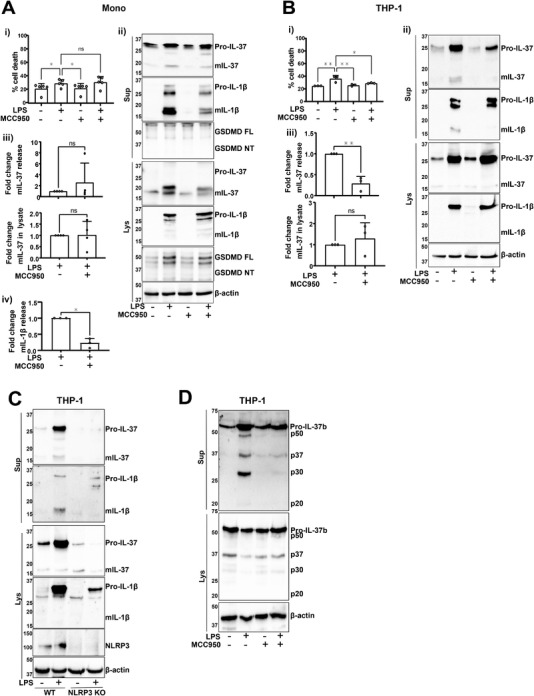
**Long‐term LPS treatment leads to IL‐37 release independent of alternative NLRP3 inflammasome**. (A) Primary human monocytes and (B) THP‐1 cells were left untreated or preincubated with MCC950 (1 μM) and stimulated with LPS (1 μg/mL, 18 h). (Ai, Bi) Cell death was measured by LDH assay and shown as percentage relative to total cell death, n = 3–5. *p < 0.05; **p < 0.01 using one‐way ANOVA. (Aii, Bii) Western blot analysis for mIL‐37 (17.5 kDa), pro‐IL‐37 (27.5 kDa), mIL‐1β (17 kDa), pro‐IL‐1β (31 kDa), GSDMD full length (FL, 53 kDa), GSDMD *N*‐terminus (NT, 31 kDa) as well as loading control β‐actin (42 kDa). (Aiii, Biii) mIL‐37 in supernatants and lysates was quantified using densitometry and standardized to LPS alone treatment. n = 3‐4. **p < 0.01 using *t*‐test. (Aiv) mIL‐1β in supernatants was quantified using densitometry and standardized to LPS alone treatment. n = 3. *p < 0.05 using *t*‐test. (C) WT or NLRP3 KO THP‐1 were left untreated or stimulated with LPS (1 μg/mL, 18 h). Western blot analysis for mIL‐37 (17.5 kDa), pro‐IL‐37 (27.5 kDa), mIL‐1β (17 kDa), pro‐IL‐1β (31 kDa), NLRP3 (113 kDa), as well as loading control β‐actin (42 kDa). (D) IL‐37b‐mCherry expressing THP‐1 cells were left untreated or preincubated with MCC950 (1 μM) and stimulated with LPS (1 μg/mL, 18 h). Anti‐IL‐37 antibody (R&D) was used to detect IL‐37b‐mCherry. All blots are representative of at least three independent biological experiments. Ns, not significant. Data are shown as mean ± SD.

Long‐term LPS treatment (18 h) has been proposed to induce the classical secretion of IL‐37 [5]. As this signal is also known to activate the alternative NLRP3 inflammasome in human monocytes [13], the role of NLRP3 for IL‐37 release in this context was investigated. To activate the alternative inflammasome, primary human monocytes were stimulated with LPS (1 μg/mL, 18 h). LPS stimulation resulted in modest but significant cell death that was not blocked by NLRP3 inhibitor MCC950 (Fig. 3Ai). Similarly, mIL‐37 release observed after LPS stimulation was not inhibited by MCC950 (Fig. 3Aii,iii), although MCC950 blocked mIL‐1β secretion as expected (Fig. 3Aii,iv). While some cell death was initiated by LPS, there was no detectable GSDMD cleavage (Fig. 3Aii).

On the other hand, long‐term LPS treatment also caused cell death of THP‐1 cells but this was inhibited by MCC950 (Fig. 3Bi) [26]. In this context, inflammasome inhibition with MCC950 (1 μM, 15 min pretreatment) blocked the release of mIL‐37 caused by LPS (Fig. 3Bii,iii). To validate the role of NLRP3 in response to LPS stimulation, this experiment was repeated in WT and NLRP3 KO THP‐1 cells. While IL‐1β processing and release only occurred in WT THP‐1s, NLRP3‐deficient cells could still produce mIL‐37 but the secretion was blocked (Fig. 3C). IL‐37b regulation in LPS‐stimulated THP‐1s was further investigated by utilizing IL‐37b‐mCherry THP‐1s. These were pretreated with MCC950 (1 μM, 15 min) and stimulated with LPS (1 μg/mL, 18 h). Similar to canonical inflammasome activation, the release of the p50, p37, and p30 fragments was observed (Fig. 3D), which were blocked by MCC950. However, the p20 fragment was not generated as following nigericin stimulation, suggesting that nigericin is activating proteases that are responsible for the generation of p20, rather than caspase‐1.

### Long‐term LPS treatment leads to IL‐37 release dependent on membrane permeability

**Figure 4:**
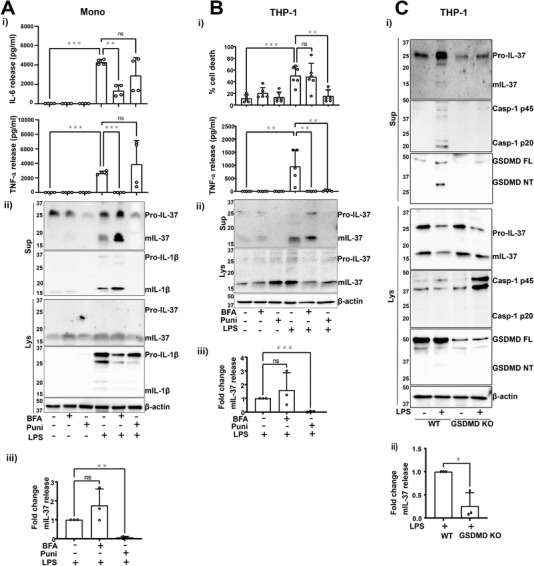
**Long‐term LPS treatment leads to IL‐37 release dependent on membrane permeability**. (A) Primary human monocytes or (B) THP‐1s were pretreated with BFA (10 μg/mL) or punicalagin (50 μM) for 15 min prior to 18‐h stimulation with LPS (1 μg/mL). (Ai) Secreted TNF‐α and IL‐6 were measured by ELISA. (Bi) Cell death was measured by LDH assay and shown as percentage relative to total cell death. n = 4–6. **p < 0.01; ***p <0.001 using one‐way ANOVA. (Aii, Bii) Western blot analysis for mIL‐37 (17.5 kDa), pro‐IL‐37 (27.5 kDa), mIL‐1β (17 kDa), pro‐IL‐1β (31 kDa), as well as loading control β‐actin (42 kDa). (Aiii, Biii) Levels of mIL‐37 release were quantified using densitometry and standardized to LPS treatment alone. n = 3. **p < 0.01; ***p < 0.001 using one‐way ANOVA. (C) WT and GSDMD KO THP‐1s were stimulated with LPS (1 μg/mL, 18 h). (Ci) Western blot analysis for mIL‐37 (17.5 kDa), pro‐IL‐37 (27.5 kDa), mCaspase‐1 (20 kDa), pro‐Caspase‐1 (45 kDa), GSDMD full length (FL, 53 kDa), GSDMD N‐terminus (NT, 31 kDa), as well as loading control β‐actin (42 kDa). (Cii) mIL‐37 release was quantified using densitometry and standardized to LPS treatment of WT cells. n = 3. *p < 0.05 using *t‐*test. All blots are representative of at least three independent biological experiments. ns = not significant. Data are shown as mean ± SD.

To further understand IL‐37 secretion in these conditions, primary human monocytes were pretreated with ER‐Golgi translocation inhibitor Brefeldin A (BFA, 10 μg/mL) to prevent classical secretion [27], or the plasma membrane stabilizing agent punicalagin (50 μM) to block membrane permeability, followed by LPS stimulation (1 μg/mL) for 18 h. LPS caused the secretion of TNF‐α and IL‐6 and as expected, BFA inhibited the release of these classically secreted cytokines [28] (Fig. 4Ai). IL‐37 immunoblotting revealed that like IL‐1β (Fig. 4Aii), prolonged LPS treatment caused the release of mIL‐37, which was inhibited by punicalagin but not BFA treatment (Fig. 4Aii,iii). Moreover, higher proportion of pro‐IL‐37 was secreted into the supernatant in the absence of treatment when cells were cultured for 18 h (Fig. 4Aii), rather than 45 min, as was the case for nigericin stimulation (Fig. 1Ai). This indicates constant and constitutive IL‐37 secretion. However, punicalagin also blocks the secretion of IL‐37 precursor, suggesting plasma membrane permeability is playing a role.

In THP‐1 cells, LPS treatment induced cell death as well as TNF‐α release (Fig. 4Bi). Punicalagin, but not BFA, blocked cell death following LPS stimulation. TNF‐α release was also inhibited by punicalagin, perhaps due to the transcriptional inhibition induced by this compound [29]. IL‐37 secretion was also observed in THP‐1s, where punicalagin but not BFA, blocked IL‐37 release as well as IL‐1β (Fig. 4Bii, iii). To uncover the role of GSDMD in IL‐37 release from THP‐1s, WT or GSDMD‐deficient cells were stimulated with LPS (1 μg/mL, 18 h). LPS treatment of WT THP‐1s resulted in GSDMD cleavage, suggesting that it may play a role in cell death and explain differences observed between primary monocytes and THP‐1 cells. GSDMD‐deficient cells showed no caspase‐1 release that was observed in WT THP‐1 cells following stimulation (Fig. 4Ci). The release of mIL‐37 was significantly reduced in GSDMD KO THP‐1s compared to WT (Fig. 4Cii).

### Mutagenesis of D20 and V46 sites does not entirely block IL‐37 cleavage

**Figure 5:**
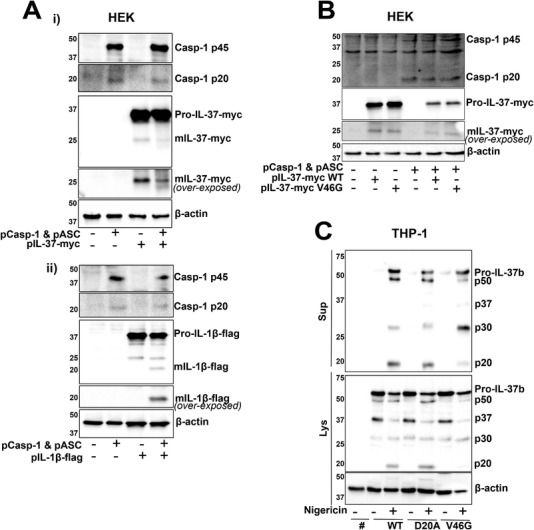
**Mutagenesis of D20 and V46 sites does not entirely block IL‐37 Cleavage**. (A) HEK293 cells were transfected with pIL‐37b‐myc/pIL‐1β‐flag with/without pCaspase‐1 and pASC. (Ai) Western blot analysis was carried out on cell lysates for Myc tag, Caspase‐1, as well as loading control β‐actin. (Aii) Western blot analysis was carried out on cell lysates for flag tag, Caspase‐1, as well as loading control β‐actin. (B) HEK293 cells were transfected with pIL‐37b‐Myc WT or V46G mutant with/without pCaspase‐1 and pASC. Western blot analysis was carried out on cell lysates for Myc tag, Caspase‐1, as well as loading control β‐actin. (C) mCherry alone (#mCherry) or IL‐37b‐mCherry WT, D20A or V46G THP‐1 cells were treated with nigericin (10 μM, 45 min). Western blotting was carried out using an anti‐IL‐37 antibody (R&D). Data shown are representative of three independent experiments.

As a caspase‐1 cleavage site has been reported, we investigated whether IL‐37b can be cleaved by active caspase‐1 in a simple HEK293 overexpression system. To address this, HEK293 cells were cotransfected with ASC and caspase‐1, which when overexpressed can autoactivate, as well as IL‐37b‐Myc or IL‐1β‐Flag. While overexpressed IL‐1β maturation occurred only in the presence of caspase‐1 and ASC (Fig. 5Aii), overexpressed IL‐37b processing (loss of 10 kDa) did not require caspase‐1, further adding to the evidence that it is cleaved constitutively (Fig. 5Ai). To investigate whether the constitutive cleavage seen is at V46 site, this site was mutated to V46G using site directed mutagenesis. V46G mutant, in the presence or absence of caspase‐1, showed the same cleavage pattern as WT IL‐37b‐Myc (Fig. 5B). The contribution of the two described cleavage sites, D20 and V46, was next assessed by mutating them to D20A as previously reported [9], as well as V46G within the lentiviral mCherry vector (Fig. 5C). Stable THP‐1 cell lines were generated to express these constructs. The D20A mutant IL‐37b‐mCherry cells displayed the same processing and release as IL‐37b‐mCherry WT cells, once again suggesting caspase‐1‐independent cleavage of IL‐37. V46G mutant, on the other hand, showed enhanced generation of the p30 fragment and decreased generation of the p50 and p20 fragments. As no difference was observed in the generation of the cleaved form of IL‐37b‐Myc V46G and WT in HEK cells, we suggest that the mutation may be impacting the processing of the mCherry tag rather than IL‐37 itself, causing additional C‐terminal processing, or is a result of a THP‐1‐specific protease.

### D20A and V46G mutants do not alter IL‐37b localization

**Figure 6:**
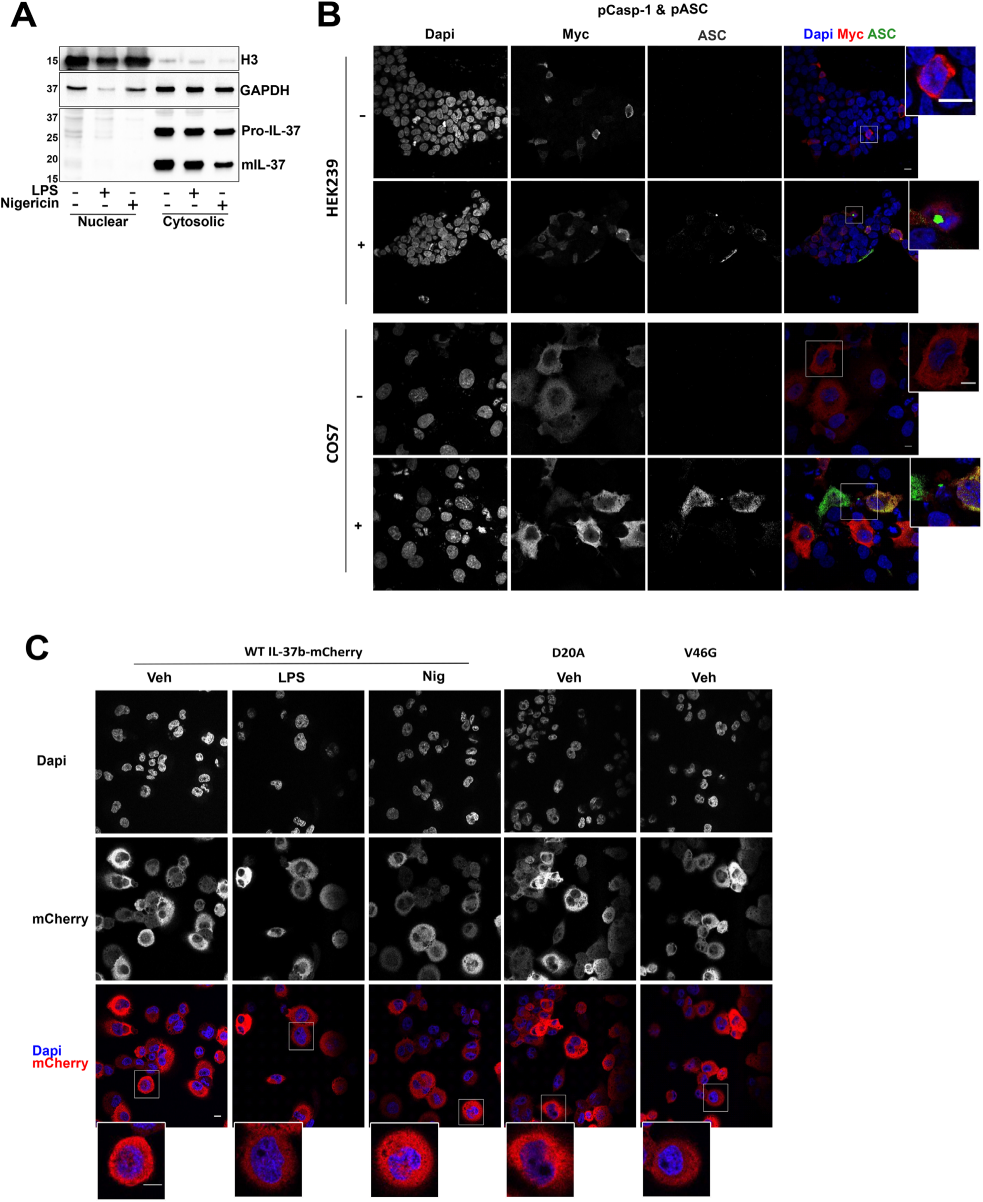
**D20A and V46G mutants do not alter IL‐37b localization**. (A) THP‐1 cells were stimulated with LPS (1 μg/mL, 18 h) or nigericin (10 μM, 45 min). Subcellular fractionation was performed. Western blot analysis for mIL‐37 (17.5 kDa), pro‐IL‐37 (27.5 kDa), histone H3 (15 kDa), and GAPDH (36 kDa). All blots are representative of at least two independent experiments. (B) HEK293 or COS7 cells were transfected with pIL‐37b‐Myc with/without pCaspase‐1 and pASC. Representative confocal images of Myc (red), ASC (green), and Dapi (blue) are shown. (C) Images from an immunofluorescence experiment showing WT/D20A/V46G IL‐37b‐mCherry overexpressing THP‐1 cells stimulated with LPS (1 μg/mL, 18 h) or nigericin (10 μM, 30 min, unprimed). Representative confocal images of mCherry (red) and Dapi (blue) are shown. Images are representative of three independent biological experiments. Scale bar is 10 μm.

The subcellular localization of transfected IL‐37 has been reported, as both cytosolic and nuclear in a RAW cell line overexpressing IL‐37b [9]. We first studied the localization of endogenous IL‐37 by subcellular fractionation. THP‐1 cells were treated with LPS (1 μg/mL, 18 h) or nigericin (10 μM, 45 min). Subcellular fractionation was carried out to obtain a nuclear fraction enriched with histone H3 and cytosolic fraction enriched with GAPDH (Fig. 6A). We found that pro‐ and mIL‐37 were both largely cytosolic. Although a very small portion of IL‐37 was nuclear, most of it was present in the cytosol and we did not find evidence for nuclear translocation upon long‐term LPS treatment or nigericin stimulation. To confirm this, IL‐37 localization was next studied by confocal microscopy. IL‐37b‐Myc was transfected into both HEK293 and COS7 cells lines in the presence or absence of pASC and pCaspase‐1, which we have shown to autoactivate (Fig. 5A). No increase in nuclear localization was observed in the presence of ASC and caspase‐1 (Fig. 6B). We next aimed to further characterize IL‐37b mutants given that the D20A mutation has been shown to prevent nuclear localization in murine RAW cells stably expressing human IL‐37. IL‐37b‐mCherry overexpressing THP‐1 cells were stimulated with LPS (1 μg/mL, 18 h) or nigericin (10 μM, 30 min). We did not observe nuclear localization of IL‐37b‐mCherry in THP‐1 cells, either at baseline or after activation with LPS (18 h) or nigericin (in the absence of priming). Moreover, neither D20A nor V46G mutants altered its cytosolic localization (Fig. 6C).

## Discussion

We found IL‐37 to be constitutively expressed and processed by caspase‐1‐independent mechanisms. Canonical NLRP3 inflammasome assembly and caspase‐1 activation were responsible for IL‐37 release in human monocytes and THP‐1 cells, but not necessarily cleavage, as the mIL‐37 form was observed in nonactive monocytes, and no changes in the mIL‐37 size were detected when a pan‐caspase inhibitor was used prior to inflammasome assembly. Processed IL‐37 has higher anti‐inflammatory potency, hence, the importance of the release of the most bioactive form in response to highly inflammatory stimuli to limit inflammation and collateral damage. This has been described for both D20 cleavage, where the mature form showed higher affinity for IL‐18Rα [7], as well as V46 cleavage, where recombinant N46‐218 IL‐37 injected into WT mice showed a larger reduction in circulating IL‐6 levels [6]. Therefore, *N*‐terminal processing, rather than the precise site of cleavage, may be important for increasing the anti‐inflammatory strength of IL‐37.

So far two different cleavage sites for IL‐37 have been proposed: one at D20 suggested to be a caspase‐1 cleavage site and another at V46, the enzyme for which remains unknown. The first report of IL‐37 (then IL‐1H) cleavage was described in transfected mammalian cell lines HEK293 and CHO [11]. The secreted pro‐ and mature forms of the protein were immunoprecipitated from the supernatant and *N*‐terminal sequencing identified that the cleaved form began at Val46. Similarly, we saw constitutive 10 kDa cleavage of IL‐37 in the physiologically relevant primary human monocytes as well as constitutive 10 kDa IL‐37b‐Myc processing in HEK293 cells. While overexpressing caspase‐1 in a HEK293 model caused IL‐1β processing, it did not induce IL‐37 processing. Kumar et al. (2002) provided the first evidence for recombinant caspase‐1‐mediated processing of IL‐37, however, they also showed maturation of exogenously expressed IL‐37 in HEK cells that lack caspase‐1, further providing evidence for caspase‐1‐independent cleavage [8]. Bulau et al. (2014) also suggested caspase‐1‐dependant cleavage of IL‐37 in LPS‐stimulated RAW macrophages, stably expressing IL‐37 [9]. They observed a mature form of IL‐37 in resting macrophages that was potentiated by LPS treatment. The generation of this LPS‐induced mature form was reduced when expressing IL‐37‐D20A mutant compared to WT. However, whether these RAW cells initiated NLRP3 inflammasome assembly and caspase‐1 activation in response to LPS was not measured, which would be important to show as most RAW cells lack ASC which is required to activate the NLRP3 inflammasome [9]. It is possible that although recombinant caspase‐1 can cleave IL‐37, in cells there are other mechanisms that regulate IL‐37 cleavage. Also, we have performed these experiments in human monocytes, which may explain differences in results compared with previous studies conducted in murine cells.

When we expressed the IL‐37(D20A)‐mCherry mutant, no changes in the constitutive cleavage were observed compared to the WT version of IL‐37, either in resting or inflammasome active cells. It is possible that this is not the bona fide caspase‐1 cleavage site. Cleavage at the predicted caspase‐1 site D20 is expected to remove just 2.2 kDa from the *N*‐terminus and could make it difficult to detect the processing by Western blot. IL‐37 is conserved in many mammals including some primates and canis [3]. Its presence in dogs, which lack functional caspase‐1 activity [30] suggests that caspase‐1 may not be required for IL‐37 processing and biological activity. In carnivorans, it has been shown that GSDMD and IL‐1β cleavage is driven by caspase‐8 instead. However, we were able to rule out the potential involvement of caspase‐8 as pan‐caspase inhibitor treatment, which was used to initiate necroptosis, and had no effects on IL‐37 cleavage. Hence, it would be necessary to further systematically address which proteases are involved. V46G mutant led to a partial reduction in the levels of p50 cleaved form of IL‐37b‐mCherry and an increase in the levels of p30 as well as a decrease in the p20 form which is only detected by the IL‐37 antibody, suggesting a C‐terminal cleavage and/or multiple rounds of processing. We still do not understand what regulates such cleavage but speculate that this is observed due to the presence of the mCherry tag, since WT and V46G mutant IL‐37b‐Myc showed the same pattern of cleavage in HEK cells in the presence or absence of caspase‐1. The appearance of this p20 form in response to nigericin (when using IL‐37b‐mCherry) does not necessarily mean that caspase‐1 is responsible for the processing, as other enzymes, such as calpains, have been described to be activated following inflammasome assembly [31]. Indeed, the p20 fragment was not generated following alternative inflammasome activation with long LPS treatment. It is also possible that more than one protease is involved in the cleavage of IL‐37, as different mature forms have been described, and that its cleavage is dependent on the inflammatory environment and triggers sensed by the cell. Further work is required to address which proteases cleave IL‐37 and the exact cleavage site.

The caspase‐1 cleavage of IL‐37 at D20 has been proposed as a requirement for IL‐37 nuclear localization [9], as D20A mutants are not able to localize to the nucleus, following LPS treatment. In our hands, we could not detect increased nuclear localization of endogenous IL‐37 after long LPS stimulation or after nigericin treatment by subcellular fractionation. Similarly, we saw no nuclear translocation of overexpressed IL‐37b‐mCherry in THP‐1 cells or IL‐37b‐Myc transfected into HEK and COS7 cell lines by confocal microscopy. The differences in localization between studies could be explained by variations in IL‐37 sequences, different cell types utilized, or different methods of subcellular fractionation used in the studies.

The release of a mature form of IL‐37 occurred in a GSDMD‐mediated manner, following canonical NLRP3 inflammasome activation [31]. IL‐37 release was blocked by punicalagin and GSDMD inhibitor NSA, but not glycine, suggesting a potential role for GSDMD pores for the release of IL‐37, as described for mIL‐1β [32]. In addition to pyroptosis, we found other lytic cell death pathways, such as necroptosis, to be a mechanism for IL‐37 release. Furthermore, following on from reports of IL‐37 production by LPS treated monocytes [5], we sought to examine the role of the alternative inflammasome. Unlike PMA differentiated THP‐1 monocytes which were found unable to assemble the alternative inflammasome [33], undifferentiated THP‐1 cells could activate caspase‐1 in response to LPS alone [26]. This was accompanied by GSDMD cleavage and a small but significant cell death. mIL‐37 release in these cells was dependent on both NLRP3 and GSDMD, perhaps as both were required for cell death. In primary human monocytes, a modest degree of cell death was also observed in response to LPS but this was not reduced by NLRP3 inflammasome inhibition. This is in agreement with past studies that described that long‐term LPS stimulated monocytes undergo apoptosis, not pyroptosis [34]. The lack of GSDMD cleavage in primary monocytes supports this theory. We have previously described a number of differences between inflammasome activation pathways in monocytes versus THP‐1 cells, including the requirement for TAK1 or NF‐κB, respectively in this pathway [19]. Therefore, it is not surprising the alternative inflammasome pathways also differ. In monocytes, mIL‐37 release, like cell death, was unaffected by blocking the alternative NLRP3 inflammasome. Plasma membrane permeability, however, was important for both pro and mIL‐37 secretion in both THP‐1 cells and primary monocytes, while inhibiting classical secretion with BFA A did not block the release of IL‐37 release in our hands. However, as the change in cell death during LPS stimulation was small, whereas the release of IL‐37 was still high compared to untreated cells, other mechanisms of nonclassical secretion may be possible. Alternative pathways involving autophagosomes, exosomes, and microvesicles have been described for IL‐1β [35]. In LPS‐stimulated monocytes, slow secretion of IL‐1β via exocytosis of secretory lysosomes was observed that was independent of GSDMD and pyroptosis [34]. For mIL‐37 release, the role of ER‐Golgi intermediate compartment vesicles has been suggested [15]. Indeed, evidence for cell death‐independent secretion of IL‐37 was observed as the release of both pro and mature forms was enhanced by cell swelling caused by hypotonicity despite not increasing cell death. Hypotonicity has previously been reported to cause membrane permeability‐dependent IL‐1β secretion [20]. Hence, membrane permeability, rather than high levels of cell death is more important for IL‐37 release.

The secretion of pro‐IL‐37, but not the mature form, was observed in resting conditions and was reduced by punicalagin, suggesting that some level of plasma membrane permeability is driving pro‐IL‐37 release even in the absence of priming or inflammasome activation which enhances it. It may be the case that slow IL‐37, like slow mIL‐1β secretion, can occur through plasma membrane permeability in myeloid cells in the absence of GSDMD activation [24]. The significance of this release is not understood. It is possible that like pro‐IL‐1β, pro‐IL‐37 is released via GSDMD pores to be cleaved by other extracellular proteases, such as elastase or tryptase, secreted by immune cells like neutrophils or mast cells [36]. This would contribute to the specific inflammatory milieu of a particular inflammatory response. Also, due to the expression of IL‐37 in many different cell types, it is possible that multiple cell type‐specific enzymes are able to cleave IL‐37 [17], as is the case for IL‐1α [23]. For example, proteases, such as calpains [31], are activated downstream of NLRP3 assembly and caspase‐1‐dependent GSDMD cleavage as the proinflammatory phase progresses.

In summary, we have established novel mechanisms for IL‐37 secretion from monocytes in two distinct contexts. During rapid canonical NLRP3 inflammasome activation and pyroptosis, IL‐37 was found to be released in a NLRP3 and GSDMD manner. Necroptosis, another form of lytic cell death, also facilitated the rapid release of mIL‐37. On the other hand, during long‐term TLR4 stimulation with LPS, the secretion of IL‐37 was independent of the alternative NLRP3 inflammasome but did require plasma membrane permeability. Elevated levels of IL‐37 are a characteristic of many noncommunicable diseases [37]. For example, the NLRP3 inflammasome plays a role in the pathogenesis of rheumatoid arthritis [38]. Both IL‐1β and IL‐37 serum levels are elevated in patients with active disease [39] and correlate with severity. Thus, it makes sense that these cytokines share a mechanism of secretion. Elevated plasma IL‐37, IL‐18, and IL‐18BP levels are also observed in patients with inflammatory conditions, such as acute coronary syndrome, where NLRP3 is involved [40, 41]. Our work highlights the mechanisms by which the more potent, mature form of IL‐37 is produced by circulating monocytes to limit inflammation.

## Experimental procedures

### Reagents and cell lines

Fetal bovine serum (FBS) was obtained from Gibco. LPS (*Escherichia coli* 026:B6); nigericin sodium salt (N7143); protease inhibitor cocktail (P8340); penicillin‐streptomycin (Pen/Strep, P4333); MCC950 (PZ0280); punicalagin (P0023); glycine (G7126); Z‐VAD‐FMK (V116‐2MG), Nec‐1 (N9037), and adenosine 5′‐triphosphate disodium salt hydrate (ATP, A2383) were sourced from Sigma. NSA was purchased from Calbiochem (432531‐71‐0).

Primary antibodies for Western blot analysis and their final concentrations were: anti‐human IL‐37 (2 μg/mL, goat polyclonal, R&D Systems, AF1975), anti‐human IL‐37 (0.6 μg/mL, rabbit polyclonal, Proteintech, 11863‐1‐AP), anti‐human IL‐1β (0.1 μg/mL, goat polyclonal, R&D Systems, AF‐201‐NA), anti‐human caspase‐1 p20 (1:1000, mouse monoclonal, Cell Signalling Technology, 3866), anti‐NLRP3 (1 μg/mL, mouse monoclonal, Adipogen, AG‐20B‐0014), and anti‐β‐actin‐HRP (0.2 μg/mL, mouse monoclonal, Sigma, A3854), anti‐human GSDMD (0.07 μg/mL, Novus Biologicals, NBP2‐33422), anti‐mCherry (1 μg/mL, rabbit polyclonal, Abcam, ab167453), anti‐flag (1:1000, rabbit polyclonal, Cell Signalling Technology, 2368S), anti‐Myc (1:1000, rabbit monoclonal, Cell Signalling Technology, 2278S), anti‐Histone H3 (1:1000, rabbit polyclonal, Proteintech, 17168‐1‐AP), anti‐GAPDH (1:1000, rabbit polyclonal, Proteintech, 10494‐1‐AP). HRP‐conjugated secondary antibodies used for Western blotting were anti‐rabbit‐HRP (0.25 μg/mL, goat polyclonal, Dako, P0448), anti‐mouse‐HRP (1.3 μg/mL, rabbit polyclonal, Dako, P0260), and anti‐goat‐HRP (0.13 μg/mL, rabbit polyclonal, Sigma, A5420).

Anti‐human IL‐37 (R&D Systems, AF1975) is raised against isoform A Lys27‐Asp192 (exons 4,5,6). Anti‐human IL‐37 (Proteintech, 11863‐1‐AP) is raised against isoform B. Therefore, both antibodies are able to detect IL‐37 isoform B. R&D antibody is used to detect IL‐37 unless stated otherwise.

THP‐1, HEK293, and COS7 were purchased from ATCC. HEK293T Lenti‐X cells were purchased from Takara Bio.

### Cell culture and treatments

THP‐1 cells were cultured in RPMI‐1640 supplemented with 2 mM l‐glutamine, 10% FBS, and Pen/Strep (100 U/mL). Human PBMCs were obtained from the National Blood Transfusion Service (Manchester, UK) with full ethical approval from the Research Governance, Ethics, and Integrity Committee at the University of Manchester (ref. 2018‐2696‐5711). PBMCs were isolated from leucocyte cones from healthy donors by density centrifugation using a 30% Ficoll gradient. The PBMC layer was separated and washed with MACS buffer (PBS, 0.5% BSA, 2 mM EDTA) to remove platelets. Monocytes were positively selected from PBMCs with magnetic CD14^+^ MicroBeads (Miltenyi, 130‐050‐201) for 15 min at 4°C, and eluted using a LS column (Miltenyi, 130‐042‐401).

To carry out canonical inflammasome experiments, cells were seeded and plated at a density of 1 × 10^6^ cells/mL. Cells were primed with LPS (1 μg/mL) for 4 h in complete RPMI. The priming stimulus was then removed and replaced with ET buffer (147 mM NaCl, 10 mM HEPES, 13 mM D‐glucose, 2 mM KCl, 2 mM CaCl_2_, and 1 mM MgCl_2_). Cells were then treated with Nigericin (10 μM, 45 min) or ATP (5 mM, 1 h) to activate the NLRP3 inflammasome. The 90 mOsm hypotonic solution was achieved by diluting isotonic ET buffer (300 mOsm) 1:4 with distilled sterile water. For alternative inflammasome experiments, cells were cultured in OptiMEM‐reduced serum medium (Thermo Fisher Scientific, 11058021) and stimulated with LPS (1 μg/mL) for 18 h.

### Quantitative real‐time PCR (qPCR)

RNA was extracted from primary monocytes using the RNeasy Mini kit (Qiagen, 74104) and reverse‐transcribed to cDNA using the High‐Capacity RNA‐to‐cDNA Kit, according to the manufacturer's instructions (#4387406). IL‐37 isoform‐specific primers were obtained from Sigma. Primer sequences were taken from Ref. [42] and are as follows:

Isoform A Forward: 5’‐gcgcttaagaggtccaaaggt‐3’ Reverse: 5’‐gctatgagattcccagagtccag‐3’

Isoform B Forward: 5’‐tcacacaagtccaaaggtga‐3’ Reverse: 5’‐ agccagcttcatcagtttct‐3’

Isoform C Forward: 5’‐tcacacaaagatcttctttgca‐3’ Reverse: 5’‐cagccagcttcatcagtttc‐3’

Isoform D Forward: 5’‐gcttagaaggtccaaaggtgaa‐3’ Reverse: 5’‐ gagctcaaggatgaggctaatg‐3’

Isoform E Forward: 5’‐ gctgcttagaagagatcttcttt‐3’ Reverse: 5’‐ ctgaagggatggatgactttg‐3’

qPCR was carried out using Power SYBR Green PCR Master Mix (Applied Biosystems, 4385618) on the QuantStudio 12K Flex (Applied Biosystems). A single product was seen on melt curve analysis. The expression of two housekeeping genes was assessed using HPRT1 (QT00059066) and GNB2L1 (QT00200263) of all samples. Data were first normalised to expression levels of the housekeeping genes. Expression levels of isoforms were calculated using the 2^(‐ΔΔ^ *^Ct^* ^)^ method and are relative to isoform a.

### Cell death assay

The supernatant was centrifuged for 5 min at 500 × *g* at 4°C to remove any cells. Cell death was quantified using a colorimetric assay for the release of lactate dehydrogenase (LDH) into cell supernatants using CytoTox 96 Nonradioactive Cytotoxicity Assay (G1780, Promega), according to the manufacturer's instructions. Absorbance values were measured at 490 nm and the results were expressed as a percentage normalized to total cell lysis.

### Enzyme‐linked immunosorbent assay (ELISA)

TNF‐α (Invitrogen, #88‐7346‐86), IL‐6 (Invitrogen, #88‐7066), and IL‐1β (R&D Systems, DY201) concentration was measured in cell supernatants according to manufacturer's instructions.

### Western blot

Cells were lysed on ice using a RIPA lysis buffer (50 mM Tris‐HCl, pH 8, 150 mM NaCl, 1% NP‐40, 0.5% sodium deoxycholate, and 0.1% SDS), supplemented with a protease inhibitor cocktail (Sigma‐Aldrich, P8340, 1:100). Lysates were then centrifuged at 21 000 × *g* for 10 min to remove the insoluble fraction.

Protein concentrations of each sample were measured using BCA assay (Thermo Scientific Pierce, 23225), following the manufacturer's guidelines, to standardize the amount of protein in each sample. Cell supernatants were centrifuged at 500 × *g* for 5 min to remove cells and concentrated using 10 kDa MW cut‐off filters (Amicon, Merck Millipore), as described by the manufacturer. Supernatants and lysates were diluted in 1× reducing Laemmli buffer containing 1% β‐mercaptoethanol. Samples were heated at 95°C for 5 min and separated by tris‐glycine SDS–PAGE. Proteins were transferred onto nitrocellulose membranes (0.2 μm), followed by blocking with PBS‐Tween (0.1%) containing 5% skimmed milk for 1 h at room temperature. Membranes were then incubated overnight with the specific primary antibody in blocking buffer at 4°C. The following day, membranes were labeled with a HRP‐conjugated secondary antibody for 1 h at room temperature. Following washing, membranes were imaged using Clarity Western ECL Blotting Substrate (Bio‐Rad, 1705061) in a ChemiDoc MP Imager (Bio‐Rad). Densitometry analysis was performed using ImageJ (rsb.info.nih.gov) to measure the intensity of mIL‐37 bands. Processed IL‐37 release was compared to control treatment and expressed as fold change.

### Subcellular fractionation

Cells were lysed using a fractionation buffer containing 20 mM HEPES pH 7.4, 10 mM KCl, 2 mM MgCl_2_, 1 mM EDTA, 1 mM EGTA, 1 mM DTT, and 1:200 protease inhibitor. Samples were centrifuged at 720 × *g* for 5 min. The nuclei containing pellet was washed in fractionation buffer, centrifuged at 720 × *g* for 10 min, and resuspended in TBS with 0.1% SDS. The supernatant was centrifuged at 10 000 × *g* for 5 min. The resulting supernatant was further centrifuged at 100 000 × *g* for 1 h. This supernatant contains the enriched cytoplasmic fraction.

### Generation of IL‐37b‐cherry THP‐1 cell lines

IL‐37 isoform B was cloned from IL‐37 Human Myc‐tagged ORF clone (Origene, RC204638) which corresponds to the NM_014439 reference sequence and is referred to as variant 1 [43]. AttB overhangs were added using the following primers obtained from Sigma:

Forward: 5‘‐ggggacaagtttgtacaaaaaagcaggcttcaccatgtcctttgtgggggagaa‐3’

Reverse: 5’‐ggggaccactttgtacaagaaagctgggtcctaatcgctgacctcactgg‐3’

The PCR product was purified from the agarose gel using ISOLATE II PCR and Gel Kit (Bioline, BIO‐52059) and inserted into the donor vector (pDON‐ZEO) using BP Clonase (Invitrogen, 11789020). The resulting Entry Clone was expressed in Library Efficiency DH5α Competent Cells (Thermo Fisher Scientific, 18263012) and plated on zeocin (50 μg/mL) selective agar plates. Positive colonies were grown up and DNA isolated using QIAprep Spin Miniprep Kit (Qiagen, 27106). pLNT destination vectors were developed according to the method outlined by Bagnall et al. (2015) [44] and propagated in ccdB‐resistant bacteria (One Shot ccdB Survival 2 T1R Competent Cells, Invitrogen, A10460). The Entry Clone was inserted into the Destination Vector using LR Clonase (Invitrogen, 11791020). The Expression Clone was overexpressed in One Shot Stbl3 Chemically Competent *E. coli* (Invitrogen, C737303) and selected for using ampicillin (100 μg/mL).

Site‐directed mutagenesis was performed using QuikChange II XL Site‐Directed Mutagenesis Kit (Agilent Technologies, 200521). The following primers were obtained from Sigma:

D20A Forward: 5’‐gcagcactggggtt**cgg**ctttttcccagtcctcag‐3’

D20A Reverse: 5’‐ctgaggactgggaaaaag**ccg**aaccccagtgctgc‐3’

V46G Forward: 5’‐tcacctttggacttgtgtg**ccc**aaaattcatggcggggag‐3’

V46G Reverse: 5’‐ctccccgccatgaatttt**ggg**cacacaagtccaaaggtga‐3’

HEK293T Lenti‐X cells were plated at 4 × 10^5^ per well in a six‐well plate. Lipofectamine 2000 was used following the manufacturer's instructions. Briefly, 8 μL lipofectamine, 1.2 μg pMD2.G, 0.4 μg psPAX2, and 1.5 μg of the generated IL‐37b‐mCherry vector were used per reaction to transfect HEK293T cells. The following day, the media were replaced, and cells were incubated for 2 more days. Supernatants were filtered with a 0.45 μm to remove any contaminating cells. Supernatants containing viral particles expressing our vector of interest were used to transduce 5.0 × 10^4^ THP‐1 cells using 8 μg/mL polybrene. THP‐1 cells, together with both the viral particles and polybrene, were centrifuged at 1000 × *g* for 1 h at 30°C. The pellet was resuspended in fresh complete RPMI and plated in a 12‐well plate. The cell line was then sorted for mCherry‐positive cells using a BD Influx cell sorter (top 50% mCherry expressing population).

### HEK293 and COS7 transfection

HEK293 and COS7 cells were cultured in complete DMEM; 5 × 10^4^ cells were plated on coverslips. The following day, transfection was carried out with Lipofectamine 2000 following the manufacturer's instructions. A total of 0.5 μg of each vector was used per reaction to transfect HEK cells. The following day, the media were replaced and cells were incubated for an additional day. pASC was kindly gifted by Dr. Pablo Pelegrin (IMIB‐Arrixaca, Murcia) and pCaspase‐1 by Prof. Jürg Tschopp.

### Immunofluorescence

THP‐1 cells were plated on glass coverslips with 0.05 μM PMA (Sigma‐Aldrich, P1585) and treated as described. HEK293 cells were plated on poly‐l‐lysine‐coated coverslips (Sigma‐Aldrich, P8920). COS7 cells were plated directly on coverslips and transfected as described above. Cells were fixed with 4% paraformaldehyde in 1× PBS for 15 min and permeabilized with 0.1% triton X‐100 while blocking with 2% BSA in PBS. Anti‐Myc (Cell Signalling Technology, #2278) and anti‐ASC (BioLegend, 676502) were used at 1:300 and 1:1000, respectively, overnight at 4°C. Coverslips were then washed five times in PBS and incubated with Donkey anti‐Rabbit AF647 (Thermo Fisher Scientific, A31573) and Donkey anti‐mouse AF488 (Thermo Fisher Scientific, A21202). Coverslips were washed with PBS as before. A final wash in distilled water was carried out before drying and mounting onto a glass slides using Dako Fluorescence Mounting Medium (Agilent Technologies, S3023). Images were collected on a Leica SP8x AOBS Inverted confocal using a 60× oil objective. Images were analyzed using ImageJ (rsb.info.nih.gov).

### Mass spectrometry

THP‐1 cells stably expressing IL‐37b‐mCherry were stimulated as described in ET buffer. The supernatant was concentrated by using Amicon Ultra‐15 Centrifugal Filter Unit (UFC9010). Immunoprecipitation of mCherry tagged protein was carried out using RFP‐Trap Magnetic Agarose (Chromotek, rtma) according to manufacturer's instructions. Samples were ran on an SDS‐PAGE gel and stained or destained using Coomassie Brilliant Blue solutions (BioRad, 1610436 and 1610438). Bands of interest were excised from the gel and dehydrated using acetonitrile followed by vacuum centrifugation. Dried gel pieces were reduced with 10 mM DTT and alkylated with 55 mM iodoacetamide. Gel pieces were then washed alternately with 25 mM ammonium bicarbonate followed by acetonitrile. This was repeated, and the gel pieces were dried by vacuum centrifugation. Samples were digested with elastase for 2 h at 37°C. Digested samples were analyzed by LC‐MS/MS using an UltiMate 3000 Rapid Separation LC (Dionex Corporation, Sunnyvale, CA) coupled to an Orbitrap Elite (Thermo Fisher Scientific, Waltham, MA) mass spectrometer. Peptide mixtures were separated using a gradient from 92% A (0.1% FA in water) and 8% B (0.1% FA in acetonitrile) to 33% B, in 44 min at 300 nL/min, using a 75 mm × 250 μm i.d. 1.7 mM BEH C18, analytical column (Waters). Peptides were selected for fragmentation automatically by data‐dependent analysis. Data produced were searched using Mascot (Matrix Science UK), against the (Swissprot and Trembl) database with taxonomy of [human] selected. Data were validated using Scaffold (Proteome Software, Portland, OR).

### Statistical analysis

GraphPad Prism 9 software was used to carry out all statistical analysis. Data were first tested for normality using Shapiro–Wilk test. Differences between 3+ groups were analyzed using one‐way ANOVA with the *post‐hoc* Dunnett's test or two‐way ANOVA with the *post‐ hoc* Tukey's test for multiple comparisons. Data was shown as mean ±SD. Accepted levels of significance were *p < 0.05, **p < 0.01, ***p < 0.001, ****p < 0.0001. ns, not significant.

We thank Inés Díaz‐del‐Olmo (Lydia Becker Institute of Immunology and Inflammation, Manchester) for generating the GSDMD KO THP‐1 cells, Prof. Veit Hornung (Ludwig Maximilian University, Munich) for gifting the NLRP3 KO THP‐1 cells, Dr. Kevin Stacey and Judit Gali Moya (Lydia Becker Institute of Immunology and Inflammation) for their support in isolating primary human cells and Dr. Gareth J. Howell (University of Manchester Flow Cytometry Core Facility) for his help with FACS. The Bioimaging Facility microscopes used in this study were purchased with grants from BBSRC, Wellcome, and the University of Manchester Strategic Fund. Special thanks goes to Darren Thomson for help with the microscopy. We also thank Biological Mass Spectrometry Facility (BioMS) of the University of Manchester (Dr. David Knight and Emma Keevill).

## Conflict of interest

The authors A.G., R.D‐P. and G. L‐C. report no conflict of interest.

## Ethics approval statement

The manuscript does not contain experiments using animals or human studies.

## Author contributions

A.G. and G.L‐C. designed research; A.G. and R.D‐P. performed research; A.G and R.D‐P. analyzed data; A.G. and G.L‐C. discussed data; G.L‐C. supervised the project, A.G. and G. L‐C. wrote the paper.

### Peer review

The peer review history for this article is available at https://publons.com/publon/10.1002/eji.202149724.

**Abbreviations**
DAMPs: damage‐associated molecular patterns
ERGIC: ER‐Golgi intermediate compartment
FBS: fetal bovine serum
GSDMD: gasdermin‐D
LDH: lactate dehydrogenase
mIL‐37: mature IL‐37
NLRP3: NOD‐like receptor pyrin domain‐containing protein 3
Nec‐1: necrostatin‐1
NSA: necrosulfonamide
Pen/Strep: penicillin‐streptomycin
RFP: red fluorescent protein

## Supporting information

Supporting Information

## Data Availability

All data relevant to the study are included in the article or as supplementary information.
