# Supplementary material for: NLRP3 inflammasome triggers interleukin‐37 release from human monocytes
Source: Eur J Immunol. 2022 Apr 28;52(7):1141–57. doi: 10.1002/eji.202149724 (PMC9540663; doi:10.1002/eji.202149724)
Supplement: Supplementary file 1 — Supporting Information [file EJI-52-1141-s001.pdf]

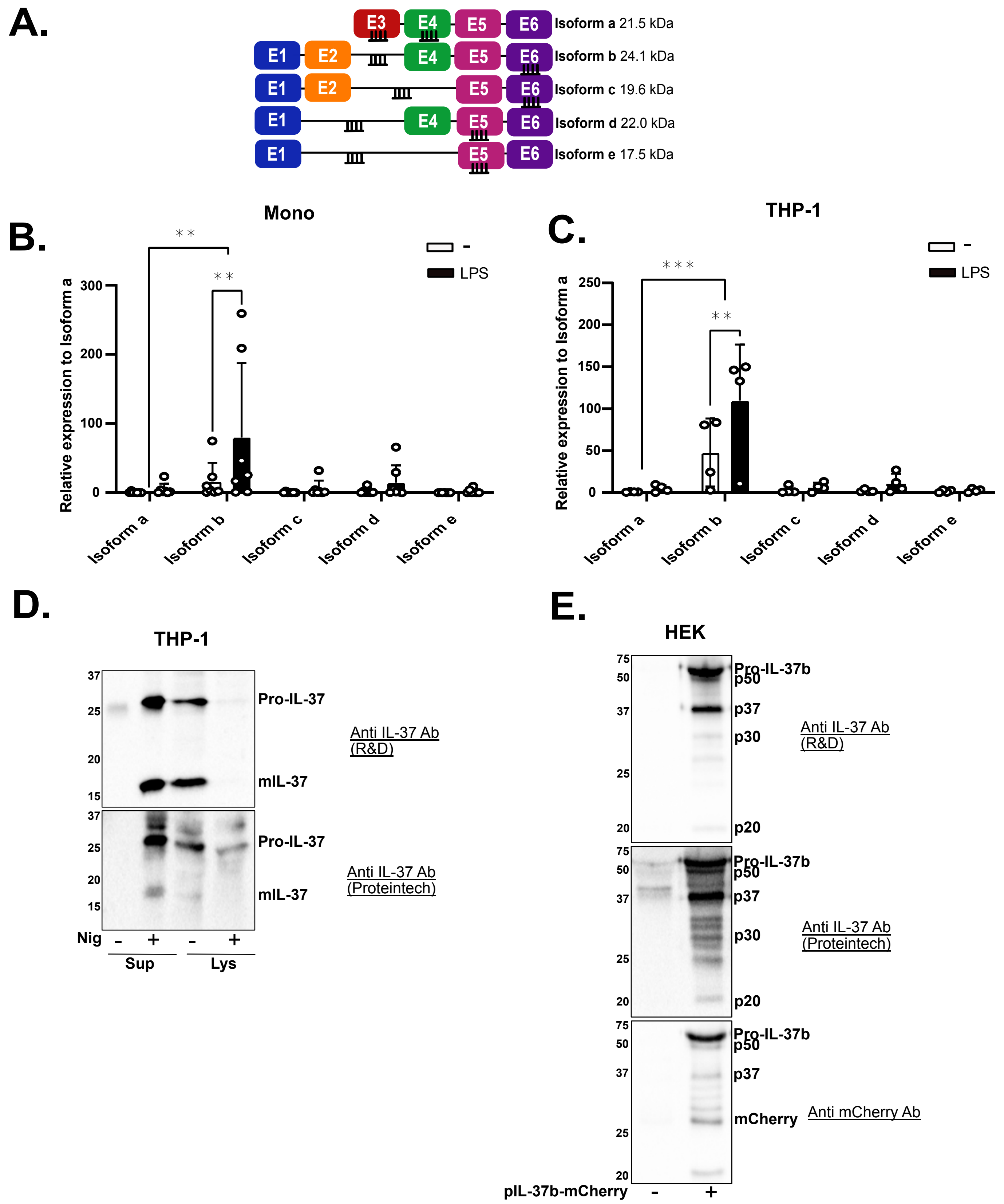

Figure 1S: IL-37 isoform B is dominant in Human Monocytes and is recognised by R&D and Proteintech Antibodies (A) Alternative splicing generates 5 IL-37 isoforms of various sizes. Markers show the binding sites for isoform specific primers. (B) Quantitative RT-PCR showing the mRNA expression levels of IL-37 isoforms in primary human monocytes in untreated or LPS (1 µg/mL, 4h) treated cells. IL-37 expression was standardised to housekeeping genes HPRT1 and GNB2L1. Bars represent the mean fold increase of each isoform relative to basal levels of IL-37 isoform A ± S.D., n=7 (Biologically independent), **P < 0.01 using a two-way ANOVA. (C) Quantitative RT-PCR showing the mRNA expression levels of IL-37 isoforms in THP-1 cells in the presence/absence of LPS (1 µg/mL, 4h). Ct values were standardised to housekeeping genes HPRT1 and GNB2L1. Bars represent the mean fold increase from IL-37 isoform A ± S.D., n=4 (Biologically independent), **P < 0.01, *** = P <0.001 using a two-way ANOVA. Data is shown as mean +/- S.D. (D) THP-1s cells were treated with nigericin (10 µM, 45 min). Western blot analysis for mIL-37 (17.5 kDa), pro-IL-37 (27.5 kDa). (E) HEK293 cells were transfected with pIL-37b-mCherry. Blots were carried out using R&D and Proteintech antibodies and are representative of 2 independent experiments.

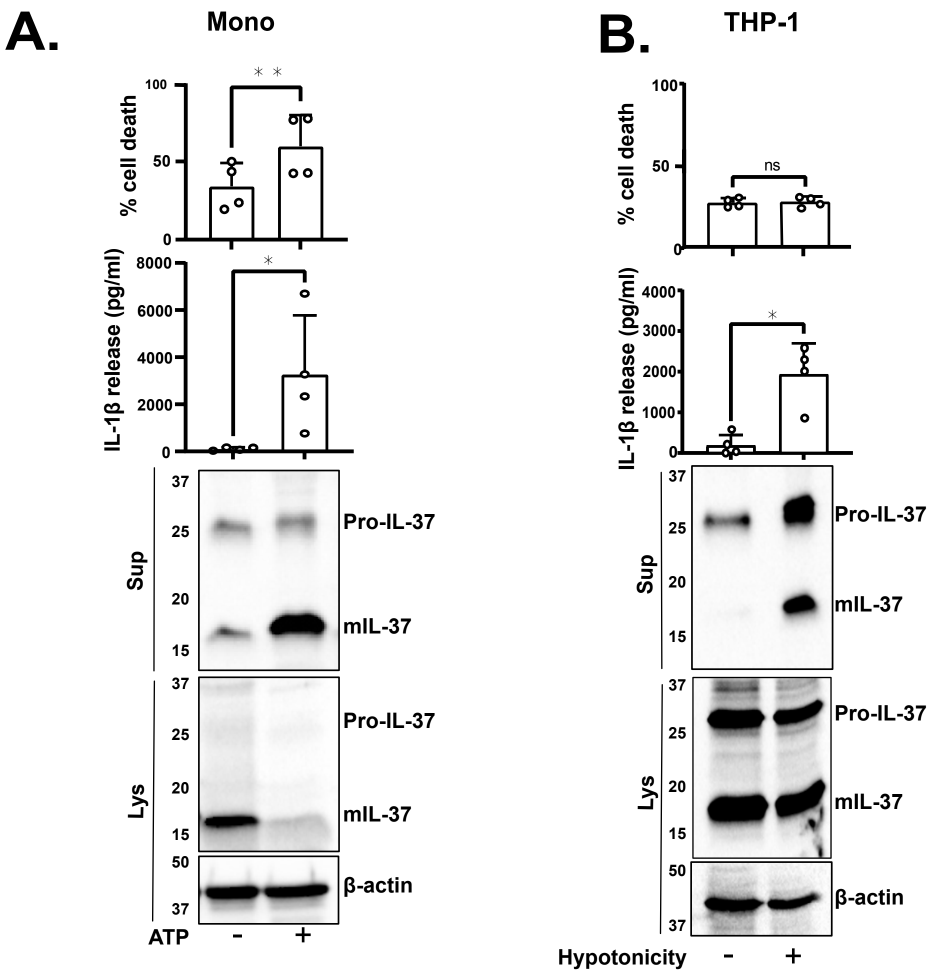

Figure 2S: NLRP3 Inflammasome activation with extracellular ATP and Swelling releases mIL-37
(A) Primary human monocytes were primed with LPS (1 µg/mL, 4h) and left untreated or stimulated with ATP (5 mM, 1h). Cell death was measured by LDH assay and shown as percentage relative to total cell death, n=4. Western blot analysis for mIL-37 (17.5 kDa), pro-IL-37 (27.5 kDa) as well as loading control β-actin (42 kDa). Secreted IL-1β was measured by ELISA, n=4. * = $P < 0.05$; ** = $P < 0.01$. (B) THP-1 cells were primed with LPS (1 µg/mL, 4h). Media was then changed to hypotonic solution. Cell death was measured by LDH assay and shown as percentage relative to total cell death, n=4. Secreted IL-1β was measured by ELISA, n=4. * = $P < 0.05$. Western blot analysis for mIL-37 (17.5 kDa), pro-IL-37 (27.5 kDa) as well as loading control β-actin (42 kDa). Blots are representative of 2 independent experiments. ns = not significant. Data is shown as mean +/- S.D.
